# GA-HPO PPO: A Hybrid Algorithm for Dynamic Flexible Job Shop Scheduling

**DOI:** 10.3390/s25216736

**Published:** 2025-11-04

**Authors:** Yiming Zhou, Jun Jiang, Qining Shi, Maojie Fu, Yi Zhang, Yihao Chen, Longfei Zhou

**Affiliations:** 1 College of Artificial Intelligence and Robotics, Hunan University, Changsha 410082, China; zym911@hnu.edu.cn; 2 College of Mechanical and Vehicle Engineering, Hunan University, Changsha 410082, China; junjiang@hnu.edu.cn; 3 School of Geosciences and Info-Physics, Central South University, Changsha 410083, China; stongshi74@gmail.com; 4 School of Automation Science and Engineering, South China University of Technology, Guangzhou 510641, China; au_maojiefu@mail.scut.edu.cn; 5 College of Information Science and Engineering, Northeastern University, Shenyang 110819, China; 20224084@stu.neu.edu.cn; 6 College of Physics and Optoelectronic Engineering, Shenzhen University, Shenzhen 518116, China; surewinhulk@gmail.com; 7 School of Engineering, University of North Florida, Jacksonville, FL 32224, USA

**Keywords:** hybrid algorithm, genetic algorithm, proximal policy optimization, dynamic flexible job shop scheduling problem, reinforcement learning, hyperparameter optimization

## Abstract

The Job Shop Scheduling Problem (JSP), a classical NP-hard challenge, has given rise to various complex extensions to accommodate modern manufacturing requirements. Among them, the Dynamic Flexible Job Shop Scheduling Problem (DFJSP) remains particularly challenging, due to its stochastic task arrivals, heterogeneous deadlines, and varied task types. Traditional optimization- and rule-based approaches often fail to capture these dynamics effectively. To address this gap, this study proposes a hybrid algorithm, GA-HPO PPO, tailored for the DFJSP. The method integrates genetic-algorithm–based hyperparameter optimization with proximal policy optimization to enhance learning efficiency and scheduling performance. The algorithm was trained on four datasets and evaluated on ten benchmark datasets widely adopted in DFJSP research. Comparative experiments against Double Deep Q-Network (DDQN), standard PPO, and rule-based heuristics demonstrated that GA-HPO PPO consistently achieved superior performance. Specifically, it reduced the number of overdue tasks by an average of 18.5 in 100-task scenarios and 197 in 1000-task scenarios, while maintaining a machine utilization above 67% and 28% in these respective scenarios, and limiting the makespan to within 108–114 and 506–510 time units. The model also demonstrated a 25% faster convergence rate and 30% lower variance in performance across unseen scheduling instances compared to standard PPO, confirming its robustness and generalization capability across diverse scheduling conditions. These results indicate that GA-HPO PPO provides an effective and scalable solution for the DFJSP, contributing to improved dynamic scheduling optimization in practical manufacturing environments.

## 1. Introduction

The Dynamic Flexible Job Shop Scheduling Problem (DFJSP) is recognized as a core challenge in intelligent manufacturing, due to its task heterogeneity, resource allocation uncertainty, and disturbances from dynamic events. Since being proven NP-hard in 1993 [1], its computational complexity has driven extensive research. Metaheuristic algorithms remain the primary approaches to address this problem, including the Genetic Algorithm (GA) [2,3,4,5,6], Tabu Search [7,8,9], Ant Colony Optimization (ACO) [10,11], and Particle Swarm Optimization (PSO) [12,13]. Among these, GA is widely applied owing to its simplicity, adaptability, and robustness. Since Holland’s introduction of the fundamental theorem of Genetic Algorithms [14], the schema theorem has provided a theoretical basis for their global optimization capability. More recently, GA has been combined with deep reinforcement learning to enhance adaptability under dynamic conditions [15,16]. In parallel, several studies have formalized scheduling as a Markov Decision Process (MDP) [17]. Within this framework, reinforcement learning (RL) has demonstrated strong potential for dynamic scheduling. Policy-based methods are particularly promising, as they can directly search for optimal policies in continuous action spaces, while supporting stochastic strategies. Parameter updates are typically realized through either gradient-based or gradient-free approaches [18]. Among them, policy gradient (PG) methods dominate, with REINFORCE [19] serving as the earliest Monte Carlo policy gradient algorithm and forming the foundation of subsequent advanced RL approaches.

Here, we propose GA-HPO PPO, a novel hybrid algorithm that systematically integrates Genetic Algorithms (GAs), Hyperparameter Optimization (HPO), and Proximal Policy Optimization (PPO) to address the Dynamic Flexible Job Shop Scheduling Problem (DFJSP). The main contributions of this work are threefold: Firstly, we employ a GA to automatically evolve and optimize the neural network architecture of the PPO agent, leveraging its global search capability to identify high-performing model structures. Secondly, we incorporate Optuna-based HPO to fine-tune the reinforcement learning hyperparameters, enabling adaptive and efficient policy training. Third, we integrate these optimized components into the PPO framework to achieve real-time, dynamic scheduling that accommodates stochastic task arrivals and machine heterogeneity. By combining these methodologies, GA-HPO PPO aims to minimize the number of tardy tasks, reduce the makespan, and improve machine utilization in dynamic environments, providing a robust and scalable solution for complex industrial scheduling scenarios.

To achieve these objectives, this study seeks to answer the following research questions:

- How can a hybrid algorithm be effectively constructed to leverage the complementary strengths of Genetic Algorithms (GAs) for global search and Proximal Policy Optimization (PPO) for adaptive policy learning, specifically for the DFJSP?
- To what extent can the proposed GA-HPO PPO algorithm improve key scheduling performance metrics—such as the number of tardy tasks, makespan, and machine utilization—compared to established benchmarks like DDQN, standard PPO, and rule-based heuristics?
- Does the integration of automated hyperparameter optimization (HPO) using Optuna contribute significantly to the stability, convergence speed, and final performance of the reinforcement learning agent in dynamic scheduling environments?
- How well does the trained GA-HPO PPO model generalize across diverse and unseen scheduling scenarios, demonstrating robustness to variations in problem scale and dynamics?

Our paper presents an AI-driven scheduler (GA-HPO PPO) for the Dynamic Flexible Job Shop Scheduling Problem (DFJSP) in smart manufacturing environments. The scheduler operates using real-time shop-floor data collected from heterogeneous sensors, including PLC/MTConnect for machine status, RFID and vision systems for job arrivals, and power, vibration, and temperature sensors for machine health monitoring, as well as queue length and buffer sensors. In practice, the policy processes these sensor-derived states to generate dispatching decisions online. We demonstrate how sensor-integrated production lines can apply our method to reduce overdue tasks, minimize makespan, and improve machine utilization in real time. The proposed method integrates multi-source sensor streams into a compact scheduling state, and employs GA-based hyperparameter optimization to enhance policy robustness under sensor noise and stochastic arrival events. Designed for deployment in Industrial IoT and edge computing environments near machine controllers and robotic cells, the algorithm responds effectively to asynchronous sensor events. To support reproducibility, we provide comprehensive experimental details, code, and datasets that emulate live sensor streams, including arrival events and machine status signals.

The remainder of this paper is organized as follows: Section 2 reviews optimization literature for the Flexible Job Scheduling Problem (FJSP) and introduces the fundamental theories of Probabilistic Inference Optimization (PPO) and genetic algorithms. Section 3 formally defines the Dynamic Flexible Job Scheduling Problem (DFJSP) and presents its mathematical model. Section 4 elaborates on the proposed genetic algorithm-PPO hybrid algorithm, including its components and workflow. Section 5 describes the experimental setup and dataset, followed by a comprehensive performance analysis. Finally, Section 6 summarizes the research findings and outlines future research directions.

## 2. Literature Review

### 2.1. Optimization Methods for the FJSP

Recent years have witnessed extensive research focused on the Flexible Job Shop Scheduling Problem (FJSP), widely recognized as a time-consuming and challenging domain. Demonstrated to be NP-hard [20], obtaining optimal FJSP solutions within reasonable timeframes is often infeasible. While global optimization algorithms, notably Genetic Algorithms (GAs) and Particle Swarm Optimization (PSO), exhibit significant advantages for the FJSP, they exhibit limitations in addressing dynamic scheduling scenarios.

The integration of reinforcement learning (RL) with improved global optimization algorithms has emerged as a prominent research trend. Xu et al. [21] integrated Quantum Particle Swarm Optimization (QPSO) and Variable Neighborhood Search (VNS) into a hybrid HQPSO-VNS algorithm for effective FJSP resolution. Fan et al. [22] proposed a hybrid Jaya algorithm incorporating Tabu Search to minimize makespan in the FJSP. Zhang et al. [23] developed an algorithm combining Graph Neural Networks (GNNs) and Proximal Policy Optimization (PPO) for the VPT-FJSP, achieving superior performance. Guo et al. [24] introduced an improved Genetic Programming Hyper-Heuristic (GPHH) method for the DFJSP, balancing solution efficiency and effectiveness.

Algorithm hybridization, combining multiple algorithms to leverage their strengths and mitigate their weaknesses, produces Hybrid Algorithms (HAs) known for their versatility, robustness, and effectiveness in complex optimization [25,26,27,28]. Examples include Ding et al.’s [29] HLO-PSO for FJSP; Xie et al.’s [30] hybrid GA and Tabu Search for distributed FJSP; Chen et al.’s [31] self-learning GA (SLGA), integrating SARSA and Q-learning with a GA to enhance efficiency; and Zhang et al.’s [32] graph neural network modeling and RL method for adaptive job-shop scheduling.

Despite these improvements, hybrid approaches retain limitations. Research specifically targeting the optimization of dynamic and flexible shop-floor scheduling remains relatively scarce. Challenges include the requirement for real-time scheduling, insufficient model adaptability to environmental uncertainty, and the complexity arising from numerous optimization objectives, collectively restricting a thorough exploration of the search space [33]. Consequently, susceptibility to local optima persists, creating the potential for premature convergence [34].

In summary, distinct algorithms possess inherent strengths and weaknesses for the FJSP. Researchers should carefully consider algorithm-specific characteristics and pursue deeper integration to leverage complementary advantages, thereby tailoring methodologies specifically for the FJSP to achieve enhanced scheduling performance.

### 2.2. PPO Algorithm

Proximal Policy Optimization (PPO) is a reinforcement learning algorithm designed to optimize policies, while maintaining stability during updates. The fundamental principle of PPO is to constrain the step size of policy updates, thereby preventing drastic changes that could destabilize the learning process. A key feature of PPO is the use of a “clipped loss function”, which regulates the policy updates. During each update, the algorithm computes the probability ratio between the new and old policies, and restricts this ratio within a predefined range (typically [1−ε,1+ε], where ε is a small constant). This constraint ensures that the new policy does not deviate excessively from the previous one, thereby preserving stability and preventing the policy from entering an unstable state [35].

The PPO algorithm comprises five steps. First, interaction data, including the state, action, reward, and potentially the next state, are collected by executing the current policy within the environment. Second, the relative advantage of each action is estimated using a dominance function, which may be customized or set to the default function in the high-level API of TensorFlow Agents. Third, a specially designed objective function is employed to iteratively update both the old and new policies. Fourth, policy parameters, denoted as θ, are updated using the gradient ascent method. Finally, these steps are repeated with updated policy parameters until certain stopping criteria, such as stabilized policy performance or a predefined number of iterations, are met.

### 2.3. Genetic Algorithm

The Genetic Algorithm (GA) is an optimization algorithm inspired by the process of natural evolution. It evolves an optimal solution from an initial population of solutions through operations such as selection, crossover, and mutation. The basic principles of the GA are as follows [36,37,38]:

- First, an initial population containing multiple individuals is generated. Each individual represents a potential solution to the problem. The fitness value of each individual in the population is calculated, where the fitness function evaluates the “survivability” of each individual based on its performance.
- Individuals with higher fitness are more likely to be retained and used to generate the next generation. To potentially produce better solutions, selected individuals are paired to exchange some of their genes, generating new individuals.
- Then, the newly generated offspring undergo random mutations in some genes to introduce new gene combinations, increasing population diversity and preventing the search from becoming trapped in local optima.
- The newly generated offspring replace part or all of the old population, forming a new generation. The steps of fitness evaluation, selection, crossover, and mutation are repeated until the termination condition is met.

### 2.4. Our Research Content and Objectives

This study introduces GA-HPO PPO, a novel algorithm integrating Proximal Policy Optimization (PPO), Genetic Algorithms (GAs), and Optuna-based hyperparameter optimization to address the Dynamic Flexible Job Shop Scheduling Problem (DFJSP). The approach implements real-time scheduling, accounting for task-specific attributes (arrival time, deadline, type) and heterogeneous machine processing capabilities.

GA-HPO PPO is comparatively evaluated against Double Deep Q-Networks (DDQNs), standard PPO, and conventional rule-based methods to demonstrate its efficacy in dynamic scheduling scenarios. The task scheduling performance, generalization capability, and robustness of models trained with PPO versus GA-HPO PPO are rigorously assessed.

Additionally, the static variant of the problem (without dynamic arrivals) is treated as a global optimization problem. Here, a GA derives theoretically optimal scheduling plans for baseline comparison.

The research objectives are as follows: (1) validate GA-HPO PPO’s superior convergence rate and scheduling solution quality; (2) demonstrate the hybrid algorithm’s enhanced capability for pursuing near-optimal scheduling in real-world production environments; and (3) minimize tardy task count, reduce makespan (total completion time), and improve machine utilization, thereby increasing scheduling process efficiency.

This work contributes advanced algorithmic solutions toward developing intelligent production management systems.

### 2.5. Summary of Previous Work

To provide a clearer overview of the landscape of existing research, Table 1 summarizes the key contributions and limitations of the main related works discussed above, and highlights the position of our proposed method.

## 3. Problem Description

### 3.1. Dynamic Flexible Job-Shop Scheduling Problem

Dynamic Flexible Job Shop Scheduling Problems (DFJSP) differ fundamentally from traditional flexible job shop scheduling problems, with their defining characteristic being their dynamic nature. Unlike conventional problems, where all tasks are known in advance, the DFJSP involves tasks arriving over time, each with unique arrival times and deadlines. This dynamic nature necessitates real-time adjustments to scheduling strategies within limited timeframes, to handle task uncertainties and allocate resources flexibly. Moreover, dynamic environments require simultaneous optimization of multiple objectives, such as minimizing delayed tasks, reducing total completion time, and maximizing machine utilization, all while adhering to complex constraints. These challenges place high demands on the efficiency and adaptability of scheduling algorithms, making the DFJSP a particularly intricate and demanding problem to solve.

**Figure 1:**
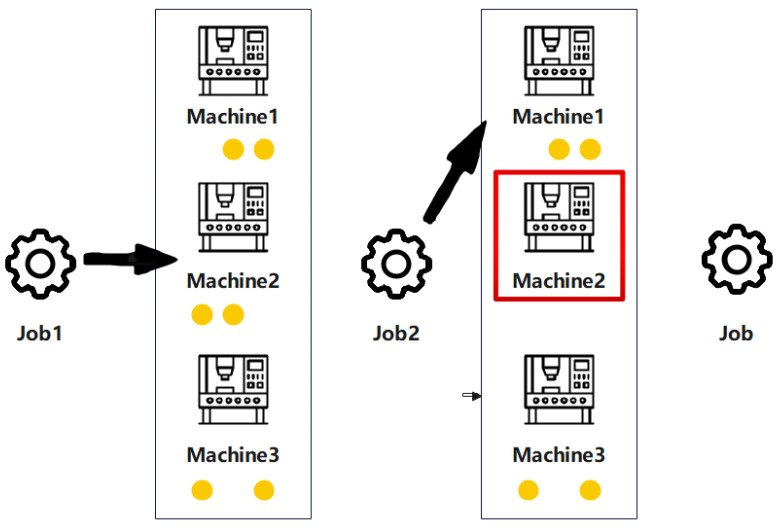
The workflow of task scheduling.

As illustrated in Figure 1. The yellow dots represent the job numbers that can be completed. The red boxes indicate that the machine is in operation. When a new task arrives, the system must select a machine that is both compatible and available. Each machine is capable of processing specific types of tasks, represented by the yellow circles labeled with corresponding task type indices. A red border around a machine indicates that it is currently occupied and cannot be assigned new tasks. In such cases, the arriving task must either be assigned to another suitable machine that is free or wait until the current machine becomes available. This visualization underscores the real-time decision-making and resource constraints inherent in the DFJSP [39].

### 3.2. Problem Formulation

The DFJSP involves scheduling the execution of n jobs on m machines. Each job has its own arrival time, deadline, and task type, and the arrivals are dynamic, with each operation requiring the selection of a machine from the set of available machines. At moment zero, all jobs and machines are available and each machine can only execute one operation at a time. It is worth noting that since preemption is not allowed, each operation, once started, must be completed without interruption. The DFJSP is machine-dependent because the processing time of each operation is different on any allowed machine and the types of tasks that can be processed by different machines are different. Its main goal is to assign each operation to a suitable machine and to sequence the operations to minimize the job interval (i.e., the total time required to complete all jobs). In Table 2, we label all the notations required for mathematical modeling and use mathematical formulas to express the environmental constraints and scheduling rules for the flexible shop floor job scheduling problem.

The following are specific descriptions of the two decision variables:

$$x_{ik}=\left\{\begin{array}{cc} 0 & \mathrm{if}\;\mathrm{task}\;i\;\mathrm{is}\;\mathrm{assigned}\;\mathrm{to}\;\mathrm{machine}\;k\\ 1 & \mathrm{otherwise} \end{array}\right.$$

$$U_{i}=\left\{\begin{array}{cc} 0 & \mathrm{if}\;\mathrm{task}\;i\;\mathrm{is}\;\mathrm{overdue}\\ 1 & \mathrm{otherwise} \end{array}\right.$$

In this paper, the objective function of the FJSP and the mathematical modeling formulation of the constraints are as follows:

Objective Functions:

1.   Minimize the number of tasks owed:

(1) $$U_{total}=min\sum_{i=1}^{n}U_{i}$$

2.   Minimize the total completion time

(2) $$C_{total}=min\left[max\left(C_{i}\right)\right]$$

3.   Maximize or minimize the average machine Utilization

The average utilization rate can be expressed as

(3) $$Util_{avg}=\frac{\sum_{k=1}^{m}W_{k}}{C_{total}*m}$$

When the numbers of machines and tasks are relatively small, the model prioritizes maximizing machine utilization, to lower operational costs and minimize the occurrence of overdue tasks. In contrast, when both the task volume and the number of machines are large, the machine utilization can be appropriately reduced to ensure rapid response times for task arrivals, while preventing excessive machine load. This strategy not only mitigates the risk of overloading but also extends the operating life of equipment, enabling the system to better accommodate increased scheduling demands, while maintaining cost efficiency.

Constraints:

1.   Assignment constraints

Each task must be assigned to a machine capable of processing it:

(4) $$\sum_{k\in M_{T_{i}}}x_{ik}=1$$

2.   Start and Completion Times

Ensure that each task starts after it arrives and finishes based on the processing time on the assigned machine:

(5) $$S_{i}⩾t_{i},\forall i$$

(6) $$C_{i}=S_{i}+\sum_{K=1}^{m}P_{ik}*x_{ik},\forall i$$

3.   Machine Availability Constraints

Ensure that each machine can only process one task at a time:

(7) $$S_{j}\geq C_{i}\;\forall i,j\;$$

where task i and task j are assigned to the same machine.

## 4. GA-HPO PPO Algorithm

**Figure 2:**
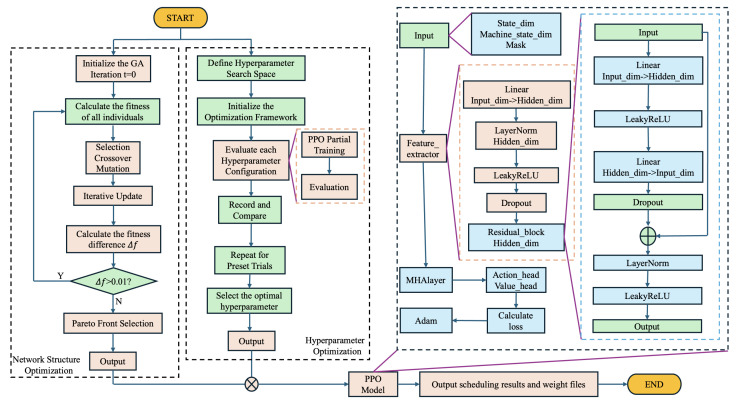
Flowchart of GA-HPO PPO.

We propose a novel hybrid algorithm that integrates Proximal Policy Optimization (PPO), a Genetic Algorithm (GA), and Optuna-based hyperparameter optimization to efficiently address the Dynamic Flexible Job Shop Scheduling Problem (DFJSP). The algorithm first utilizes a GA Optimizer module to select and optimize the neural network architecture used to make scheduling decisions. Subsequently, Optuna is employed to fine-tune the policy parameters within reinforcement learning. Based on the optimized network architecture and policy parameters, a PPO model is trained. To further enhance its scheduling performance, residual blocks and multi-head attention mechanisms are incorporated into the PPO model. The overall workflow of the algorithm is illustrated in Figure 2.

As detailed in Figure 2, the proposed model begins by leveraging the GA Optimizer and Optuna hyperparameter optimization in an iterative loop, to identify an optimal initial neural network architecture and policy parameters. This refined configuration is then passed to the PPO module for training. Within the PPO component, multi-head attention and residual blocks are introduced to strengthen feature extraction and representation learning, thereby enhancing the overall training process and final policy performance.

### 4.1. GA Optimizer

**Figure 3:**
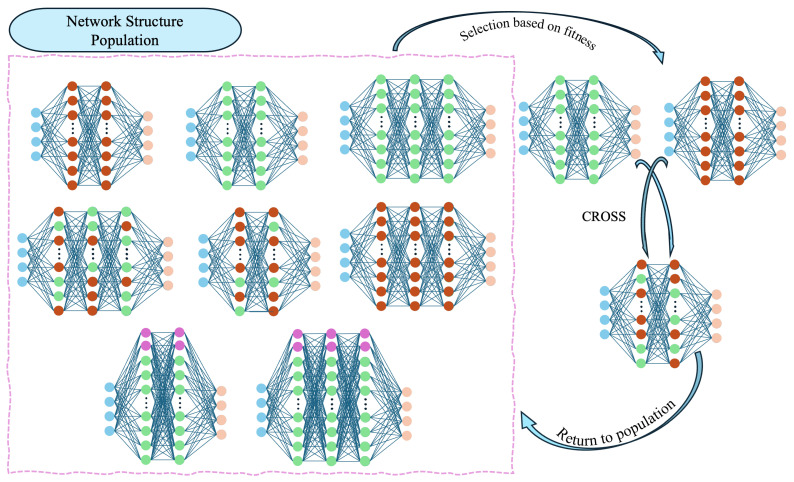
Model of GA Optimizer.

The GA Optimizer class introduces a key innovation within our hybrid framework by integrating a Genetic Algorithm (GA) to optimize the neural network architecture of the PPO ActorCritic model. The GA component applies evolutionary principles to systematically explore and iterate over different network configurations, aiming to identify architectures that enhance the performance of the reinforcement learning agent in a flexible job-shop scheduling environment. This approach allows for an adaptive search process that is well suited to the complexities of scheduling tasks, ensuring that the model can dynamically evolve to meet performance demands more effectively. The process and logic of the GA are shown in Figure 3.

#### 4.1.1. Objectives and Motivation

In reinforcement learning, the design of neural network architectures is crucial, as this directly influences the model’s ability to capture the complex patterns inherent in scheduling problems. Integrating Genetic Algorithms (GAs) into scheduling frameworks enables the automated discovery of optimal or near-optimal architectures, effectively addressing these complexities.

Conventional approaches to network architecture design often rely on manual tuning and extensive experimentation, which are both time-consuming and may fail to produce models with superior performance. In contrast, the GA provides a systematic and automated optimization mechanism, drawing on principles of natural selection to evolve network structures over successive generations. This evolutionary process accelerates convergence and enhances the model’s scheduling performance, offering an efficient and robust alternative to traditional manual design methods.

#### 4.1.2. Key Components and Workflow

**Search Space Definition:**

- Network Structures: The GA explores a predefined set of network architectures, each characterized by distinct configurations of hidden layers and neurons. This search space includes variations in layer count (e.g., two or three hidden layers) and size (e.g., 128 or 256 neurons per layer), allowing flexibility in model complexity to better suit the scheduling task.
- Feasibility and Flexibility: By operating within a structured search space, the GA ensures that exploration remains feasible and computationally manageable, balancing between thorough search and computational efficiency.

**Population Initialization:**

- Diversity: The initial population comprises a diverse set of PPO agents, each instantiated with unique network structures sampled from the defined search space. This diversity enables the GA to cover a broad spectrum of potential solutions, fostering robust exploration.
- Random Initialization: Each agent’s network parameters are randomly initialized using Xavier uniform initialization, ensuring unbiased starting conditions that support fair evolution across the population.

**Fitness Evaluation:**

- Multi-Objective Criteria: Each individual is assessed based on multiple performance metrics, including tardy task count, makespan, and machine utilization. These metrics collectively determine the fitness of each solution, reflecting its overall scheduling effectiveness.
- Training and Evaluation: The fitness evaluation method partially trains each PPO agent, evaluating performance over multiple episodes. This comprehensive assessment provides an accurate measure of each individual’s scheduling efficacy.

**Selection Mechanism:**

- Pareto Front Identification: A Pareto-based selection strategy is used to identify non-dominated individuals within the population, ensuring that only solutions excelling across all metrics are prioritized for propagation.
- Elitism: A subset of the highest-performing individuals (elite) is preserved across generations, ensuring that the top solutions are retained and have the opportunity to inform the evolution of future generations.

**Genetic Operators:**

In each generation, genetic operators are applied to introduce variation and explore the architectural search space. A crossover operation is performed between pairs of elite parent individuals. When parent architectures match, offspring parameters are created through a point-wise crossover mechanism: each parameter in the offspring network is randomly selected from one of the two parents. If the architectural structures differ, crossover is not performed directly on the parameters; instead, one parent is randomly selected and its architecture is inherited, after which the parameters are perturbed to introduce novelty. Mutation is subsequently applied with a predefined probability. The mutation operator may modify the network architecture itself—for example, by adding or removing a hidden layer, or by resizing an existing layer within predefined bounds—or may apply Gaussian noise to the model parameters to encourage local exploration. This structured approach to variation ensures that both the architectural and weight-level spaces are thoroughly explored, maintaining genetic diversity and preventing premature convergence.

To balance exploration with computational efficiency, the algorithm incorporates an early-stopping mechanism that monitors the rate of fitness improvement across a sliding window of generations. If the average improvement falls below a specified threshold, the evolutionary process is terminated early, ensuring resources are allocated efficiently, without compromising solution quality.

After the final generation, the non-dominated solutions in the population are identified to form the Pareto front, representing the best-compromise architectures across multiple objectives—such as minimizing tardy tasks and makespan, while maximizing machine utilization. These elite architectures are then advanced to the downstream hyperparameter tuning and PPO training phases. This selection mechanism ensures that only the most robust and high-performing configurations are retained, thereby enhancing the overall efficacy and reliability of the proposed scheduling framework.

### 4.2. Hyperparameter Optimization with Optuna

Hyperparameter optimization is crucial for training effective reinforcement learning (RL) agents, as hyperparameter choices significantly impact learning dynamics and performance outcomes. In this study, leveraging the optimal neural network architecture identified by the Genetic Algorithm (GA), we utilize Optuna—a powerful hyperparameter optimization framework—to fine-tune the parameters of the Proximal Policy Optimization (PPO) algorithm. This fine-tuning process aims to configure the PPO agent with optimal settings, thereby enhancing its capability to learn robust scheduling policies in a flexible job shop scheduling environment.

#### 4.2.1. Objectives and Rationale

The main objective of integrating Optuna into the PPO training pipeline is to automate and simplify the process of identifying the most efficient hyperparameter configurations. Given the huge and often unintuitive space of hyperparameters in PPO, manual tuning is neither practical nor optimal and requires a lot of time. Optuna, on the other hand, addresses this challenge by providing efficient search algorithms and intelligent sampling strategies, to discover high-performance hyperparameter sets, while reducing computational overhead.

#### 4.2.2. Hyperparameter Search Space

The choice of hyperparameters used for optimization will directly affect key aspects of the performance of the PPO algorithm. We set the range of hyperparameters for optimization to 6, the range and importance of which are listed in the Table 3.

#### 4.2.3. Optimization Process

The objective function plays a central role in Optuna’s hyperparameter optimization process, encapsulating the complete training and evaluation pipeline for each trial. In each trial, a unique set of hyperparameters are sampled from the predefined search space to initialize a PPO agent with a fixed network structure. The agent is subsequently trained in the JobShopEnv environment for a limited number of epochs to ensure computational efficiency. Following training, the agent is evaluated over multiple episodes, and the average reward obtained serves as the primary performance metric. This metric is returned as the optimization target, guiding Optuna toward hyperparameter configurations that maximize agent performance.

Optuna initiates a study with the objective of maximizing the average reward, thereby enhancing scheduling performance. The study executes a predetermined number of trials (e.g., 50), systematically exploring the hyperparameter space using advanced sampling techniques such as the Tree-structured Parzen Estimator (TPE) to achieve efficient and comprehensive search.

For each trial, the sampled hyperparameters are used to train and evaluate the PPO agent, with performance recorded based on the average reward. These trial outcomes enable Optuna to iteratively refine its sampling strategy, progressively converging toward hyperparameter configurations that yield superior performance.

Upon completion of all trials, Optuna identifies the hyperparameter set that achieved the highest average reward. This optimal configuration is subsequently employed to reinitialize the PPO agent for more extensive training, leveraging both the network architecture optimized via the Genetic Algorithm and the finely tuned hyperparameters.

Through this methodology, the PPO agent undergoes a targeted optimization process in which each phase contributes to improved scheduling performance. This approach underscores the synergistic advantage of combining a robust search strategy with intelligent hyperparameter tuning.

### 4.3. PPO Actor–Critic Model

The PPO Actor–Critic model is a central component of the Proximal Policy Optimization (PPO) algorithm, functioning as both a policy and value function approximator within a reinforcement learning framework. This dual-purpose network is designed to address the complexities of the dynamic flexible job shop scheduling problem. It integrates advanced architectural features, including residual blocks, multi-head attention mechanisms, and strategic weight initialization, to improve both the convergence rate and scheduling performance of the model.

#### 4.3.1. Architectural Components

**Feature Extractor:**

The feature extractor is a modular, sequential architecture designed with a dynamic construction to flexibly adjust to the varying levels of complexity required by different tasks. Built according to a predefined network structure, this design dictates the number and size of hidden layers, allowing the model to adaptively balance capacity and computational efficiency. Each hidden layer incorporates a linear transformation, followed by layer normalization, Leaky ReLU activation, dropout for regularization, and a residual block. This composition ensures robust feature extraction, mitigating against issues such as vanishing gradients and promoting stable learning dynamics across layers.

**Residual Blocks:**

Implemented through the ResidualBlock class, the residual connections within the network aim to improve the gradient flow, thereby facilitating the training of deeper architectures. Each residual block is structured with two linear layers, with a Leaky ReLU activation function applied between them, followed by dropout and layer normalization. The skip connection between input and output enables the network to learn both identity mappings and more complex transformations as needed, effectively supporting the network’s capacity to perform intricate feature extractions, while maintaining model stability.

**Multi-Head Attention Layer:**

**Figure 4:**
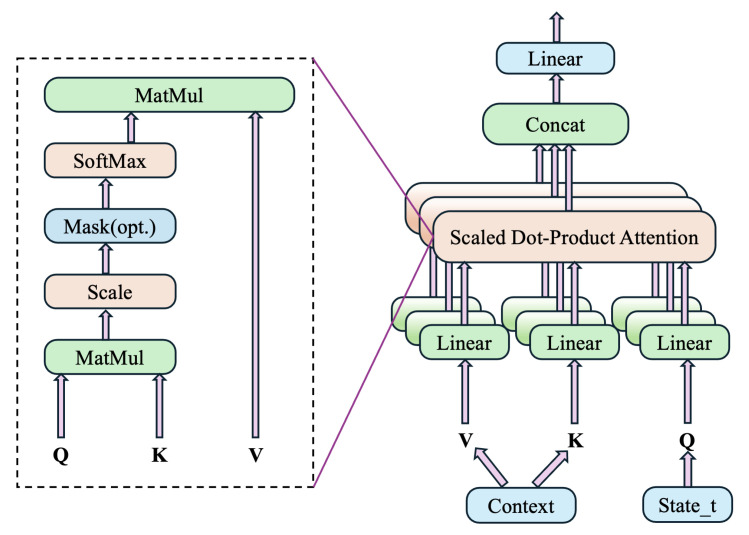
Flowchart of multi-head attention layer.

The multi-head attention mechanism, integrated through the MHAlayer class, enhances the model’s ability to capture interdependencies and interactions within the input data, particularly between machine states and task contexts. This layer processes the normalized state vector as the query and the machine context as keys and values, calculating attention scores that dynamically adjust the weighting of input features. By selectively focusing on relevant parts of the state and context, the attention layer provides the network with the ability to refine its focus based on specific scheduling scenarios, ultimately improving the accuracy and relevance of the model’s decisions. The specific logic is shown in Figure 4.

**Action and Value Heads:**

The model includes two distinct output heads: an action head and a value head. The action head is a linear layer that outputs logits corresponding to each possible action, which are subsequently converted into probabilities via a softmax function, enabling action selection based on learned policies. Conversely, the value head estimates the value of the current state, offering a baseline for advantage calculation in Proximal Policy Optimization (PPO) updates. This dual-head configuration ensures that the model can simultaneously assess action quality and optimize policy performance, supporting robust decision-making in dynamic scheduling tasks.

#### 4.3.2. Weight Initialization

The PPO Actor–Critic model adopts Xavier uniform initialization for all linear layer weights and initializes biases to zero, ensuring stability and efficiency during training. This initialization approach facilitates consistent signal propagation across layers, preventing gradient vanishing or explosion issues and promoting faster convergence. Proper weight initialization is crucial for stable training dynamics and enhances the model’s ability to effectively learn from early stages.

### 4.4. PPO Algorithm

The PPO (Proximal Policy Optimization) class encapsulates a reinforcement learning framework designed for task scheduling applications. This implementation aims to optimize scheduling performance by increasing machine utilization, reducing late tasks and maximizing scheduling efficiency. The class exploits key aspects of the PPO algorithm such as dominance estimation, policy shearing, and entropy regularization to achieve stable and efficient policy optimization.

#### 4.4.1. Initialization of the PPO Class and Key Hyperparameters

The PPO class is initialized with several hyperparameters critical for stable and effici- ent learning:

a. State Dimension (state_dim) and Action Dimension (action_dim):
  Represent the number of features in the task space and the number of available machines.
b. Network Structure (network_structure):
  Defines the neural network architecture through a series of hidden layers, controlling network depth and learning capacity.
c. Learning Rate (lr) and Discount Factor (γ):
  Regulate the speed of gradient updates and the weighting of future rewards, respectively.
d. Clipping Parameter (eps_clip):
  Limits the magnitude of policy updates, thus preventing drastic policy changes and ensuring stable learning.
e. Update Epochs (K_epochs):
  Specifies the number of times the policy network is updated in each learning cycle.
f. GAE Parameter (λ):
  Used in Generalized Advantage Estimation (GAE) to balance bias and variance in advantage estimation.
g. Batch Size (batch_size):
  Controls the size of mini-batches for each update, which directly impacts the stability and speed of learning.

#### 4.4.2. Generalized Advantage Estimation (GAE) Calculation

The GAE (Generalized Advantage Estimation) method is used to compute the advantage at each time step, which improves stability by reducing the variance in advantage values. The calculation is as follows:

(8) $$\delta_{t}=r_{t}+\gamma \cdot V\left(s_{t+1}\right)-V\left(s_{t}\right)$$

where

- $r_{t}$ is the reward received at time step *t*.
- $V(s_{t})$ and $V(s_{t+1})$ are the estimated values of states $s_{t}$ and $s_{t+1}$, respectively.
- γ is the discount factor that determines the importance of future rewards.

Using the temporal difference error $\delta_{t}$, the advantage $A_{t}$ at each time step is computed recursively as

(9) $$A_{t}=\delta_{t}+\left(\gamma \cdot \lambda \right)\cdot A_{t+1}$$

where

- λ is a smoothing parameter that controls the balance between bias and variance in the advantage estimation.

This recursive calculation produces a sequence of advantages, which the policy network uses to make more accurate updates based on both immediate and future rewards.

#### 4.4.3. Policy Update

To prepare data for training, the stored states, machine states, actions, rewards, and other information in the Memory buffer are converted into tensors. This step ensures that the data are in a suitable format for efficient computation within the network. Following data preparation, the actor–critic network is used to compute the state values, and the GAE (Generalized Advantage Estimation) method is applied to calculate the advantage values. To improve training stability, the calculated advantages are then normalized, reducing potential sources of instability.

The policy update in the PPO algorithm involves using a clipped objective function to prevent excessively large updates, which can destabilize training. The clipped policy objective is defined as

(10) $$L^{\mathrm{CLIP}}\left(\theta \right)=E_{t}\left[\min\left(r_{t}\left(\theta \right)\cdot A_{t},\mathrm{clip}\left(r_{t}\left(\theta \right),1-\epsilon ,1+\epsilon \right)\cdot A_{t}\right)\right]$$

where

- $r_{t}\left(\theta \right)=\frac{\pi_{\theta }\left(a_{t}|s_{t}\right)}{\pi_{\theta_{\mathrm{old}}}\left(a_{t}|s_{t}\right)}$ is the probability ratio between the new and old policies.
- ϵ is the clipping parameter that limits the range of policy updates.
- $A_{t}$ is the estimated advantage for each action.

The clipping operation ensures that the policy update does not deviate too far from the previous policy, maintaining stable learning. The overall objective function combines the clipped policy loss, value loss, and an entropy bonus to encourage exploration:

(11) $$L=L^{\mathrm{CLIP}}\left(\theta \right)-c_{1}\cdot E_{t}\left[{\left(V\left(s_{t}\right)-V_{\mathrm{target}}\right)}^{2}\right]+c_{2}\cdot E_{t}\left[H\left(\pi_{\theta }\left(\cdot |s_{t}\right)\right)\right]$$

where

- $c_{1}$ and $c_{2}$ are coefficients that weight the value loss and entropy term, respectively.
- $V_{\mathrm{target}}$ is the target value, typically computed as $V_{\mathrm{target}}=A_{t}+V\left(s_{t}\right)$.
- $H\left(\pi_{\theta }\left(\cdot |s_{t}\right)\right)$ represents the entropy of the policy, promoting exploration by penalizing deterministic policies.

This combined loss function contributes to stabilizing the training process, while simultaneously facilitating policy improvement and ensuring adequate exploration.

## 5. Experiments

This section uses two DFJSP datasets to assess the effectiveness of hybrid strategies and evaluate the performance of GA-HPO PPO.

### 5.1. Problem Instances and Parameter Setting

All algorithms were implemented in Python 3.10 and executed on a MacBook Pro equipped with an M3 chip running macOS. The GA-HPO PPO model was trained on four datasets with varying scales, ranging from 100 × 5 to 1000 × 30 (n × m, where n denotes the number of tasks and m denotes the number of machines). Detailed descriptions of the tasks and machines are provided in Table 4 and Table 5. To evaluate the generalization capability of the trained model, a test set comprising 10 datasets—widely recognized as benchmarks in the literature on Dynamic Flexible Job Shop Scheduling Problems (DFJSP)—was employed.

For performance comparison, GA-HPO PPO was benchmarked against a Genetic Algorithm (GA), Proximal Policy Optimization (PPO), Double Deep Q-Network (DDQN), and the combined FCFS + EAMS algorithm. The specific parameter settings for all methods are summarized in Table 6. The parameter configuration of GA-HPO PPO was determined based on the V1 dataset.

### 5.2. Environment Setup

In the code implementation of GA-HPO PPO, JobShopEnv is a tailored environment developed to model complex Dynamic Flexible Job Shop Scheduling Problems (DFJSP) within the OpenAI Gym framework. This environment integrates key elements typical to such scenarios, including tasks, machines, service times, and scheduling constraints. The primary objective of JobShopEnv is to provide a practical and interactive experimental framework, enabling Reinforcement Learning (RL) agents to optimally learn and refine scheduling strategies.

#### 5.2.1. State and Action Spaces

The state representation is formulated as a high-dimensional vector that comprehensively encodes the current status of all machines, together with additional engineered features. Specifically, it comprises machine states, including the current processing times of each machine normalized by the maximum service time, and additional features, encompassing statistical descriptors such as the average and standard deviation of machine busy times, the current time, makespan, the number of tardy tasks, machine utilization, and load factors. All features are individually normalized to ensure consistent scaling, thereby facilitating more effective learning.

The action space is defined as discrete, where each action corresponds to selecting a machine for processing the current task. The total number of actions equals the number of available machines. To address infeasible decisions, such as assigning a task to an incompatible machine, JobShopEnv incorporates an action masking mechanism that dynamically invalidates actions based on task requirements and machine availability.

#### 5.2.2. Reward Function

A reward function is a short-term reward for taking an action in the current state. In RL, there is no labeling of objective values as in supervised learning, so the reward function is crucial for guiding algorithm training and should satisfy the following three principles: Firstly, the computation of the reward function should be realized instantly, as a way to evaluate the goodness of the agent’s current action and to guide the next move; Secondly, the definition of the reward function should involve long-term considerations, i.e., the computation of the reward function should take into account the goodness or badness of the actor’s current action; Thirdly, the definition of the reward function should entail long-term considerations, and the maximization objective function and the maximum expected cumulative reward should be consistent in order to achieve the ultimate goal; Finally, the design of the reward function should be generalized, considering not only the general situation in the environment, but also certain special situations in the environment.

This paper set the number of overdue tasks, total completion time, and machine utilization as optimization objectives. By employing a weighted sum approach with each objective assigned a weight summing to 1, along with various other constraints, we successfully achieved a balance among them:

- **Late Penalty:** A negative reward is given if a task is completed after the deadline, the size of which is controlled by the parameter alpha.
- **Duration penalties:** Penalize extended completion times (makespan) to encourage efficient scheduling.
- **Machine Utilization Reward:** Reward high machine utilization to promote efficient use of available resources.
- **Dense Reward Structure:** Combines smooth transitions and nonlinear scaling to provide continuous feedback and promote more stable and efficient learning by the RL agent.

The following is a formulaic representation of the specific implementation:

(12) $$utilization\_reward=\gamma *Util_{avg}$$

(13) $$makespan\_penalty=-\beta *C_{total}$$

(14) $$tardiness\_penalty=-\alpha *U_{total}$$

γ,β,α are three independent variables that we set to directly weigh the priorities of the three objective optimizations.

#### 5.2.3. Environment Dynamics

The step function controls the dynamics of the environment and determines how actions are translated into state transitions and rewards. The key process consists of three elements: Firstly, each successful task assignment updates the current time, busy time, and other state vectors of the assigned machine to ensure that all characteristics are accurately reflected, so that the next task can successfully find an available machine. Secondly, it keeps track of scheduled and skipped tasks and manages the task queue and arrival times to ensure that all tasks are scheduled.

### 5.3. Results and Analysis

#### 5.3.1. Convergence Performance

**Figure 5:**
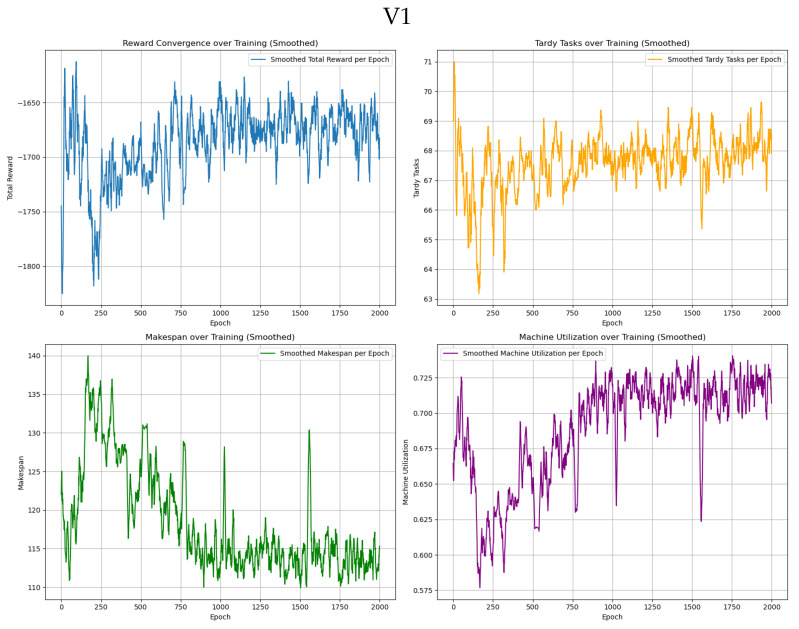

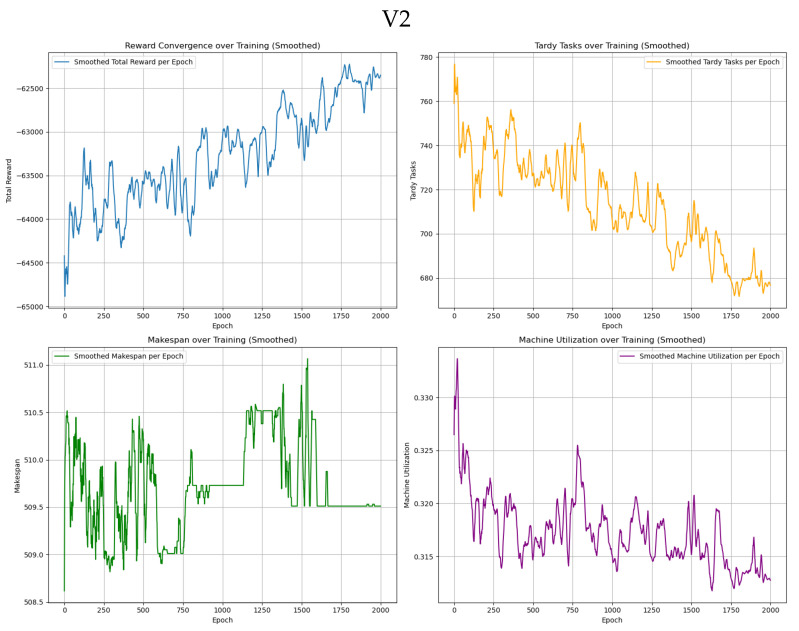
Convergence curve of PPO.

**Figure 6:**
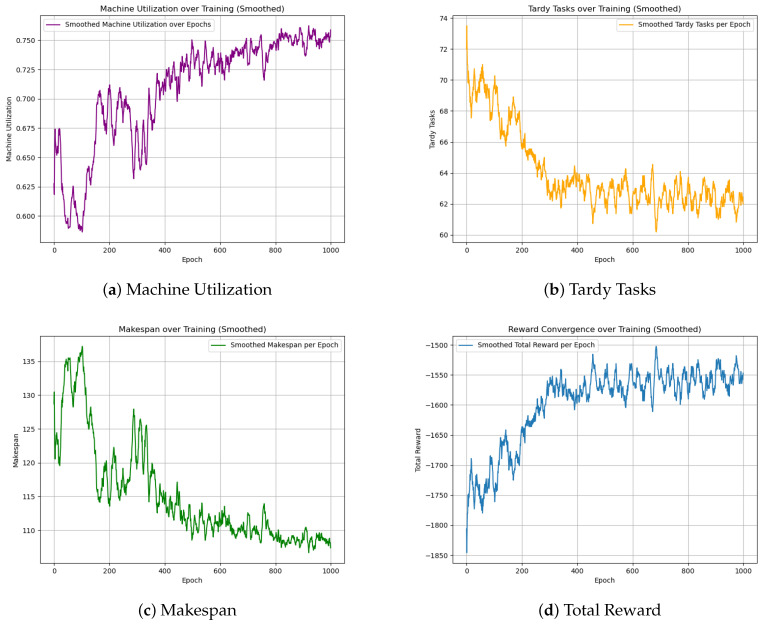
Convergence curve of GA-HPO PPO V1.

**Figure 7:**
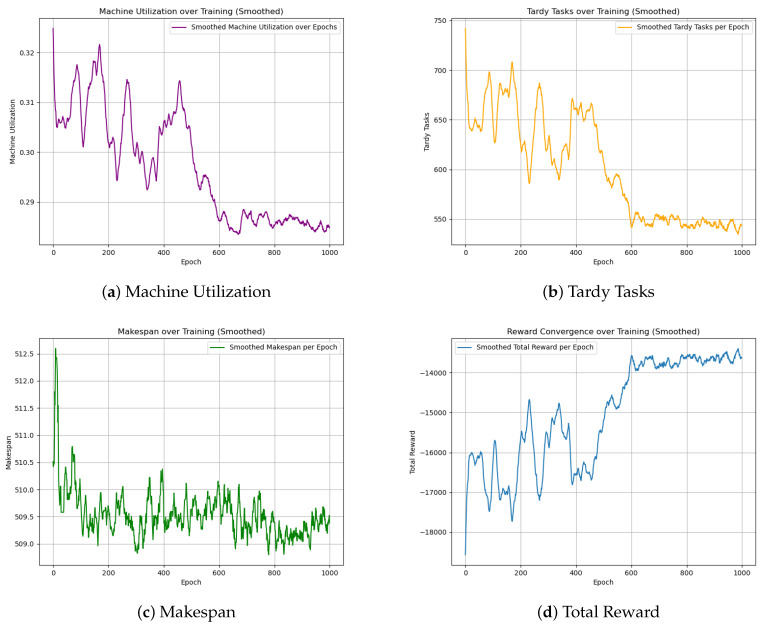
Convergence curve of GA-HPO PPO V2.

The GA-HPO PPO and PPO algorithms were trained on four datasets: two with dimensions of 5 × 100, and two with dimensions of 30 × 1000. Each dataset was trained for 2000 iterations using the PPO algorithm and 1000 iterations using the GA-HPO PPO algorithm. For both algorithms, convergence curves (Figure 5, Figure 6 and Figure 7) were obtained for the total reward, number of overdue tasks, total completion time, and machine utilization. Using datasets V1 and V2 as examples, the PPO algorithm demonstrated a convergence trend; however, it was less distinct and exhibited greater variability, indicating instability during training. In contrast, the GA-HPO PPO algorithm achieved convergence at approximately 600 iterations, despite using only half the number of training generations, and the training curve stabilized around 1000 iterations. These results highlight the superior convergence speed and stability of the GA-HPO PPO algorithm.

#### 5.3.2. Comprehensive Comparison of the Metrics of the Five Algorithms and Visualization Results

Table 7 presents the optimal performance metrics across four datasets for the five evaluated algorithms. Analysis reveals that the Genetic Algorithm (GA), as a global optimization method, approached near-optimal solutions when dynamic mechanisms were disabled.

For 100-task scheduling, GA-HPO PPO significantly outperformed the traditional reinforcement learning methods (PPO, DDQN) in minimizing tardy tasks. The algorithm simultaneously maintained a high machine utilization, ensuring timely task completion, while optimizing the machine load.

For 1000-task scheduling, GA-HPO PPO demonstrated enhanced capability in tardy task minimization. It achieved strategic resource allocation through moderated machine utilization and stabilized makespan. This indicated an effective reduction in task delays and completion times, while preserving response capacity for stochastic task arrivals.

**Figure 8:**
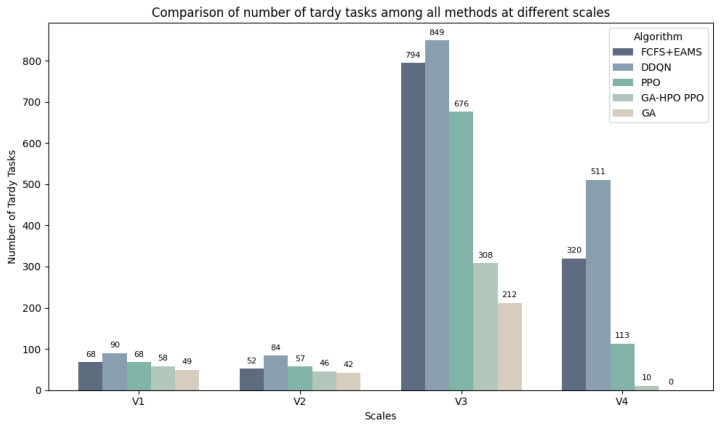
Comparison of the number of tardy tasks.

The critical metric of total tardy tasks is visualized in Figure 8 via a bar chart, providing a clear comparative analysis of the algorithmic performance.

Collectively, GA-HPO PPO demonstrated robust scheduling capabilities and stability across both the 100-task and 1000-task scenarios, establishing its effectiveness for reinforcement learning-based solutions to dynamic flexible job shop scheduling problems.

Based on the V1 and V2 datasets, each of the five algorithms were executed 20 times, and the results were comprehensively analyzed. The performance comparison revealed that GA-HPO PPO demonstrated superior performance in machine utilization, makespan (total completion time), and minimization of tardy tasks, indicating significant advantages in resource allocation and task efficiency. Compared to the benchmark algorithms, GA-HPO PPO maintained high machine utilization across the diverse conditions, effectively reducing idle time.

GA-HPO PPO also excelled in makespan minimization, consistently achieving the shortest makespan among all algorithms. In addition, it yielded the fewest tardy tasks, demonstrating exceptional deadline adherence, while the benchmark methods such as FCFS-EAMS and DDQN exhibited higher tardiness counts, indicating limitations in time- sensitive scheduling.

Overall, GA-HPO PPO generally outperformed the benchmarks and demonstrated a notable reduction in tardy tasks across the varied scenarios, while also maintaining competitive performance in completion time and resource utilization. This balance establishes its suitability for practical scheduling applications that require efficiency, resource conservation, and time optimization. In contrast, FCFS-EAMS consistently underperformed in both makespan and tardiness, particularly in complex or dynamic environments. The GA achieved competitive makespan values but exhibited higher tardiness, whereas PPO delivered a moderate performance, with less consistency across metrics.

**Figure 9:**
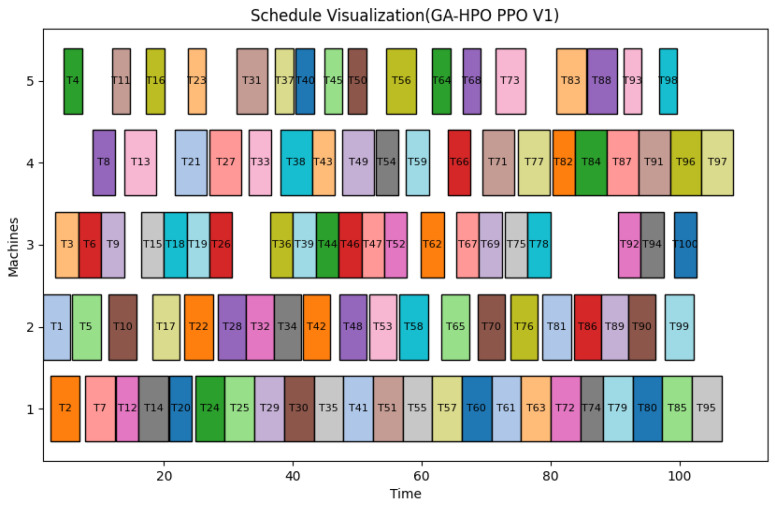
Gantt chart of GA-HPO PPO algorithm (V1).

Finally, a representative scheduling solution of GA-HPO PPO is visualized as a Gantt chart (Figure 9), providing an intuitive and clear operational timeline.

#### 5.3.3. Generalization Capability of Models

To further analyze the intelligence and stability of the agent trained by the GA-HPO PPO algorithm, we randomly generated ten additional datasets and deployed the model for task scheduling, recording relevant indicators.

In this experimental phase, we used five sets of 100-task, 5-machine data (MK01–MK05) and five sets of 1000-task, 30-machine data (MK06–MK10). As shown in the Table 8, GA-HPO PPO achieved a significantly lower number of overdue tasks compared to PPO, while maintaining or even improving on machine utilization and total completion time. Specifically, with a 100-task workload, GA-HPO PPO effectively reduced overdue tasks by about 10, and with a 1000-task workload, it achieved an average reduction of 200 overdue tasks. This improvement aligns well with industrial requirements by minimizing costs and maximizing benefits, demonstrating the robustness and generalization capability of the GA-HPO PPO model.

## 6. Conclusions

The experimental results confirmed the favorable scheduling performance and stability of the GA-HPO PPO algorithm across both 100-task and 1000-task scenarios, generally demonstrating improved performance compared to benchmark methods such as DDQN, standard PPO, and rule-based heuristics. A key strength of the GA-HPO PPO approach is its ability to notably reduce the number of tardy tasks, which were significantly lower than that of the other models evaluated. The algorithm also exhibited observable benefits in minimizing task delays, reducing makespan, and optimizing the machine load distribution, thereby supporting efficient resource utilization and timely task completion. These outcomes align with and extend the findings of earlier studies that integrated metaheuristics with reinforcement learning, such as hybrid GA-Tabu Search approaches [30] and self-learning genetic algorithms [31], through the introduction of a more adaptive and automated hyperparameter optimization framework.

However, several limitations of this study should be acknowledged. Firstly, the algorithm was evaluated using a set of predefined dynamic events and task attributes; its performance in environments with extremely high-frequency disruptions or non-stationary processing capabilities remains to be tested. Secondly, while GA-HPO PPO showed promising generalization across the benchmark datasets used, its applicability to highly specialized manufacturing contexts—such as those requiring strict precedence constraints or setup times—may require additional customization. Thirdly, the computational overhead of integrating a GA with HPO and PPO, though offset by performance improvements, may still be challenging for real-time scheduling in very large-scale systems, without further optimization or hardware acceleration.

Compared to existing approaches, GA-HPO PPO provides a balanced trade-off between solution quality and adaptability compared to traditional metaheuristics like GA or PSO, which often lack real-time responsiveness. It also shows more consistent stability and convergence behavior than pure reinforcement learning methods such as DQN and standard PPO, as observed in our experiments. Nevertheless, it should be noted that problem-specific heuristics—such as those tailored for distributed FJSP or those incorporating the problem structure via graph neural networks—may achieve competitive performance in certain constrained scenarios.

Despite these limitations, the proposed method offers a robust and scalable foundation for dynamic scheduling applications. It integrates global search capabilities with adaptive policy learning, providing a flexible framework that can be extended to incorporate additional constraints or objectives. Future work will focus on reducing computational demands, incorporating more diverse dynamic events, and validating the approach in real-world industrial settings.

## 7. Managerial Insights

The findings of this study offer several practical implications for production managers and decision-makers in manufacturing environments:

- Enhanced Responsiveness to Dynamic Events: GA-HPO PPO enables real-time adjustment to unforeseen disruptions such as machine breakdowns, urgent order insertions, or variable processing times. This capability allows managers to maintain high levels of service and on-time delivery, even under uncertainty.
- Improved Resource Utilization: By optimizing both task assignment and machine load, the algorithm helps reduce idle time and operational costs. Managers can achieve higher throughput with the same resource base, leading to better capital efficiency.
- Scalability to Large-Scale Scheduling: The algorithm’s robust performance on both small (100-task) and large (1000-task) datasets suggests its applicability in diverse industrial settings, from job shops to large-scale flexible manufacturing systems.
- Reduction in Manual Tuning Effort: The integration of automated hyperparameter optimization (HPO) and neural architecture search via the GA reduces the dependency on expert knowledge for parameter tuning, making advanced scheduling accessible to plants with limited technical expertise.
- Support for Multi-Objective Decision-Making: Managers can leverage the algorithm’s ability to balance competing objectives—such as minimizing tardiness, makespan, and maximizing utilization—to align scheduling outcomes with strategic goals like customer satisfaction, cost control, and energy efficiency.
- Facilitation of Digital Twin and Smart Manufacturing Initiatives: The reinforcement learning-based approach is compatible with digital twin frameworks and IoT-enabled production systems, providing a foundation for continuous learning and adaptive scheduling in Industry 4.0 environments.

These insights highlight how GA-HPO PPO not only advances algorithmic performance, but also delivers tangible operational benefits, supporting more agile, efficient, and intelligent manufacturing management.

## Figures and Tables

**Table 1:** Summary of contributions and limitations of key related works.

No.	Method	Key Contribution (s)	Limitation (s)
1	Genetic Algorithm (GA)	Foundation for global search in JSP/FJSP; highly adaptable via various crossover, mutation, and selection mechanisms.	Performance degrades in dynamic environments; requires full preknowledge of all tasks; convergence speed and solution quality rely heavily on parameter tuning.
2	Particle Swarm Optimization (PSO)	Effective for continuous optimization problems; faster convergence than the GA in some scenarios.	Not naturally suited for discrete scheduling problems; requires problem-specific encoding; similar to the GA, struggles with dynamic events.
3	HLO-PSO (Hybrid)	Combines human-learning concepts with PSO to enhance local search and avoid premature convergence in the FJSP.	Complex hybrid mechanism; computational overhead may be high; evaluation primarily in static settings.
4	Tabu Search	Powerful local search with memory mechanism to escape local optima.	Performance sensitive to parameter setting (e.g., tabu tenure); neighborhood search can be computationally expensive for large instances.
5	Hybrid GA-Tabu Search	Leverages a GA for global exploration and Tabu Search for intensified local search in the distributed FJSP.	Designed for a distributed and likely static environment; the integration strategy may not be directly applicable to highly dynamic DFJSP.
6	GNN + PPO	Uses Graph Neural Networks (GNNs) to effectively model shop floor state for RL in the FJSP.	Model complexity; requires structured graph representation; generalization to unseen task-machine configurations needs validation.
7	GNN + RL	Proposes an adaptive scheduling framework using a GNN to capture complex interrelationships.	Focuses on state representation; the RL agent itself may lack specialized optimization, such as HPO.
8	DQN + Rules	Integrates Deep Q-Network with composite dispatching rules to handle new job insertions in the DFJSP.	Limited by the representational capacity of predefined rules; value-based RL (DQN) may be less stable than policy-based methods (e.g., PPO).
9	SLGA (Hybrid RL-GA)	Integrates SARSA/Q-learning with a GA for self-learning evolution, improving search efficiency.	Employs simpler RL algorithms (SARSA/Q-learning) which may have limitations in complex policy learning compared to advanced policy gradient methods.
10	GPHH (Hyper-Heuristic)	Uses Genetic Programming to evolve dispatching rules for the DFJSP, offering interpretability.	Evolved rules can become complex and less interpretable; performance might plateau against non-rule-based approaches.
**This Work**	**GA-HPO PPO (Hybrid)**	**1. Integrates a GA for neural architecture search. 2. Employs Optuna for automated HPO. 3. Combines strengths of both for superior performance in the DFJSP, with demonstrated generalization.**	**1. Higher computational cost during offline optimization phase.** **2. Performance in extreme real-time constraints not yet validated.**

**Table 2:** Relevant variables and meanings.

Variable	Meaning
n	Total number of tasks
m	Total number of machines
$t_{i}$	Arrival time of task i
$d_{i}$	Deadline of task i
$T_{i}$	Type of task i
$P_{ik}$	Processing time of task i on machine k
$M_{T_{i}}$	Set of machines capable of completing task type $T_{i}$
$W_{k}$	Total time machine k is busy
$x_{ik}$	Binary variable to determine if task i is assigned to machine k
$S_{i}$	Start time of task i
$C_{i}$	Completion time of task i
$U_{i}$	Binary variable to determine if a task is overdue
$Util_{avg}$	Average machine utilization rate

**Table 3:** Hyperparameter search space.

Hyperparameter	Range	Significance
Learning Rate	[1 × 10^−5^, 1 × 10^−3^]	Determines the step size during gradient descent, affecting convergence speed and stability.
Discount Factor	[0.95, 0.99]	Balances immediate and future rewards, influencing the agent’s long-term strategy.
Clipping Range	[0.1, 0.3]	Controls the extent of policy updates, ensuring stable and conservative adjustments.
Number of Epochs	[3, 10]	Specifies how many times the entire batch of data is used for policy updates, impacting learning efficiency.
GAE Parameter	[0.9, 0.99]	Governs the trade-off between bias and variance in advantage estimation, affecting the stability of learning.
Batch Size	32, 64, 128	Determines the number of samples processed before updating the network parameters, influencing training dynamics.

**Table 4:** Task information.

Task ID	Interval Time	Arrival Time	Deadline	Task Type
1	1.24	1.24	4.47	1
2	1.13	2.37	5.95	2
3	0.81	3.19	7.11	3
…	…	…	…	…
99	0.97	97.79	101.41	2
100	1.35	99.15	104.09	3

**Table 5:** Machine information.

Service Time (Si, Tj)	Task Type 1	Task Type 2	Task Type 3
1		4.60	3.52
2	4.22	4.44	
3			3.54
4	3.56	4.90	
5	4.67		2.62

**Table 6:** All parameter settings (FCFS + EAMS is a rule-based scheduling method without any hyperparameters.)

Algorithm		Parameter Setting	
GA-HPO PPO	K_epoch = 4	lam = 0.91314	batch_size = 128
	lr = 1.1706 × 10^−5^	gamma = 0.9507	eps_clip = 0.21916
PPO	K_epoch = 10	lam = 0.95	batch_size = 64
	lr = 1 × 10^−4^	gamma = 0.99	eps_clip = 0.2
GA	Iter_max = 2000	initial_mutation_rate = 0.8	elite_rate = 0.02
	tournament_size = 5	final_mutation_rate = 0.1	
DDQN	lr = 0.0001	batch_size = 64	epsilon = 1.0
	epsilon_decay = 0.995	epsilon_min = 0.1	

**Table 7:** Data from all algorithms.

Instance	V1 (100 × 5)			V2 (100 × 5)			V3 (1000 × 30)			V4 (1000 × 30)		
	$U_{\mathrm{total}}$	$C_{\mathrm{total}}$	${\mathrm{Util}}_{\mathrm{avg}}$	$U_{\mathrm{total}}$	$C_{\mathrm{total}}$	${\mathrm{Util}}_{\mathrm{avg}}$	$U_{\mathrm{total}}$	$C_{\mathrm{total}}$	${\mathrm{Util}}_{\mathrm{avg}}$	$U_{\mathrm{total}}$	$C_{\mathrm{total}}$	${\mathrm{Util}}_{\mathrm{avg}}$
GA	49	102.62	77.04%	42	104.51	70.96%	212	508.69	22.74%	0	506.02	8.75%
PPO	68	116.90	71.20%	57	108.39	68.30%	676	509.51	31.20%	113	506.61	16.40%
GA-HPO PPO	58	108.33	75.00%	46	114.20	67.00%	308	508.62	28.30%	10	506.52	14.00%
DDQN	90	110.83	74.35%	84	113.13	70.00%	849	530.26	32.04%	511	511.34	19.52%
FCFS + EAMS	68	102.67	80.01%	52	107.56	73.91%	794	508.02	33.95%	320	506.02	20.93%

**Table 8:** Summary of PPO and GA-HPO PPO model generalization capability data.

Instance	n × m	GA-HPO PPO			PPO		
		$U_{\mathrm{total}}$	$C_{\mathrm{total}}$	${\mathrm{Util}}_{\mathrm{avg}}$	$U_{\mathrm{total}}$	$C_{\mathrm{total}}$	${\mathrm{Util}}_{\mathrm{avg}}$
MK01	100 × 5	54	114.71	72%	58	111.12	73%
MK02	100 × 5	39	116.59	63%	53	121.12	61%
MK03	100 × 5	52	106.16	77%	60	123.33	67%
MK04	100 × 5	34	109.42	62%	41	109.04	63%
MK05	100 × 5	30	102.63	68%	40	102.84	71%
MK06	1000 × 30	519	510.69	28%	684	512	31%
MK07	1000 × 30	527	503.69	29%	691	502.84	31%
MK08	1000 × 30	527	509.12	29%	704	509.53	32%
MK09	1000 × 30	578	494.46	30%	683	493.46	33%
MK10	1000 × 30	522	504.72	29%	689	506.65	32%

## Data Availability

The data that support the findings of this study are available in the main text, figures. Source data are provided with this paper. The datasets generated and/or analyzed during the current study are available from the corresponding authors on request.
